# The Palma Echo Platform: Rationale and Design of an Echocardiography Core Lab

**DOI:** 10.3389/fcvm.2022.909347

**Published:** 2022-06-21

**Authors:** Luis López, Xavier Rossello, Dora Romaguera, Ángel M. Alonso-Gómez, Estefanía Toledo, Elena Fortuny, Marta Noris, Caterina Mas-Lladó, Miquel Fiol, Raul Ramallal, Lucas Tojal-Sierra, Alvaro Alonso, Carlos Fernandez-Palomeque

**Affiliations:** ^1^Department of Cardiology, Hospital de Manacor, Manacor, Spain; ^2^Health Research Institute of the Balearic Islands (IdISBa), Palma, Spain; ^3^Department of Cardiology, Hospital Universitari Son Espases, Palma, Spain; ^4^CIBER Physiopathology of Obesity and Nutrition (CIBEROBN), Madrid, Spain; ^5^Bioaraba Health Research Institute, Osakidetza Basque Health Service, Araba University Hospital, University of the Basque Country Universidad del País Vasco/Euskal Herriko Unibertsitatea, Vitoria-Gasteiz, Spain; ^6^Department of Preventive Medicine and Public Health, University of Navarra, Instituto de Investigación Sanitaria de Navarra, Pamplona, Spain; ^7^Department of Cardiology, University Hospital of Navarra, Servicio Navarro de Salud Osasunbidea, IDISNA, Pamplona, Spain; ^8^Department of Epidemiology, Rollins School of Public Health, Emory University, Atlanta, GA, United States

**Keywords:** echocardiagraphy, core laboratory, randomized clinical trial, metabolic syndrome, atrial fibrillation, atrial function

## Abstract

**Background:**

The metabolic syndrome (MetS) is associated with increased cardiovascular morbidity and mortality. Characterization of cardiac structural and functional abnormalities due to the MetS can help recognize individuals who would benefit the most from preventive interventions. Transthoracic echocardiography (TTE) provides an opportunity to identify those abnormalities in a reproducible and cost-efficient manner. In research settings, implementation of protocols for the acquisition and analysis of TTE images are key to ensure validity and reproducibility, thus facilitating answering relevant questions about the association of the MetS with cardiac alterations.

**Methods and Results:**

The Palma Echo Platform (PEP) is a coordinated network that is built up to evaluate the underlying structural and functional cardiac substrate of participants with MetS. Repeated TTE will be used to evaluate 5-year changes in the cardiac structure and function in a group of 565 individuals participating in a randomized trial of a lifestyle intervention for the primary prevention of cardiovascular disease. The echocardiographic studies will be performed at three study sites, and will be centrally evaluated at the PEP core laboratory. Planned analyses will involve evaluating the effect of the lifestyle intervention on cardiac structure and function, and the association of the MetS and its components with changes in cardiac structure and function. Particular emphasis will be placed on evaluating parameters of left atrial structure and function, which have received more limited attention in past investigations. This PEP will be available for future studies addressing comparable questions.

**Conclusion:**

In this article we describe the protocol of a central echocardiography laboratory for the study of functional and structural alterations of the MetS.

## Introduction

Metabolic syndrome (MetS) is becoming a global public health issue (1) due to the increasing levels of obesity and physical inactivity among adults in many countries. MetS is defined by the presence of several cardiovascular risk factors (CVRFs), such as insulin resistance, dyslipidemia, and arterial hypertension, which in association with visceral obesity confer a high risk of developing cardiovascular disease (CVD) (2). From a pathophysiological perspective, it is unclear whether MetS have a similar impact on the heart and the arteries (the two components of the cardiovascular system) (3). MetS is associated with atherosclerosis and pro-thrombotic states on the arteries, whereas less is known about the impact of MetS on the function and structure of the heart (4, 5). An early detection of functional and structural myocardial alterations can be useful to better understand the association between MetS and CVD, and to identify those patients with MetS who might benefit from an early intervention (2).

Transthoracic Echocardiography (TTE) is broadly used in clinical practice to evaluate the structure and function of the heart in a non-invasive and inexpensive way (6). Moreover, TTE is a radiation-free test, available in most hospitals. A platform aimed to systematically and consistently evaluate TTEs would be useful to evaluate the underlying structural and functional cardiac substrate of participants with MetS. Thus, we report the rationale and design of the Palma Echo Platform (PEP), a platform aimed to longitudinally assess TTEs collected over a 5-year period among participants with MetS without prior CVD. This platform would be initially an opportunity to evaluate 5-year changes in the cardiac structure and function in a group of 565 individuals participating in a randomized trial of a lifestyle intervention for the primary prevention of CVD, though their use might be extended to other cardiovascular settings.

## Methods and Analysis

### Study Population

Participants have been already recruited for the PREvención con DIeta MEDiterranea-Plus (PREDIMED-Plus). This is an ongoing 6-year multicenter, parallel-group, randomized clinical trial, which is currently being conducted in 23 Spanish recruiting centers (universities, hospitals, and research institutes). Participants included women aged 60–75 years and men aged 55–75 years, without known CVD, with a body mass index (BMI) between 27.0 and 40.0 kg/m^2^ and meeting at least 3 of the following criteria defined by the International Diabetes Federation, the American Heart Association and the National Heart, Lung, and Blood Institute (4), which are: (i) elevated waist circumference (> 80 cm for women, and > 94 cm for men); (ii) elevated triglycerides (> 150 mg/dl) or drug treatment for elevated triglycerides; (iii) reduced HDL-C (< 50 mg/dL for women, and < 40 mg/dL for men) or drug treatment for reduced HDL-C; (iv) elevated blood pressure (systolic > 130 and/or diastolic > 85 mmHg) or antihypertensive drug treatment; and (v) elevated fasting glucose (> 100 mg/dl), drug treatment of elevated glucose or diabetes mellitus. This trial (ISRCTN89898870) PREDIMED-Plus aims to assess the effect of a 6-year weight loss intervention program based on an energy-restricted traditional Mediterranean diet (erMedDiet), physical activity (PA) promotion, and behavioral support, in comparison with a usual care intervention only with energy-unrestricted Mediterranean diet without any advice to increase PA or losing weight on a composite of CV events. The study protocol includes more detailed information and is available in previous publications (7) and at the website (8). All participants provided written informed consent, and the study protocol and procedures were approved according to the ethical standards of the Declaration of Helsinki by all the participating institutions.

**FIGURE 1:**
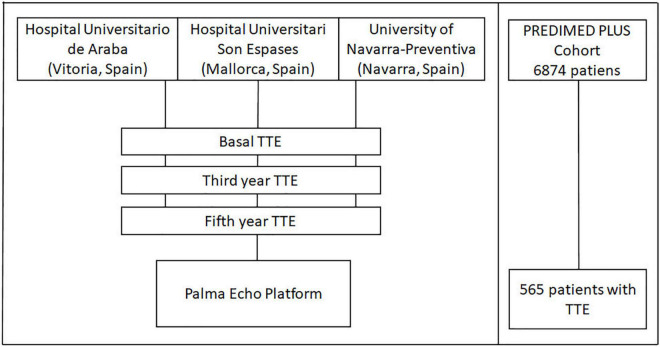
Study outline. TTE, Transthoracic echocardiography.

Among the 6,874 participants randomized between 2013 and 2016 in this trial, a sub-sample of participants from 3 recruiting centers prospectively and systematically underwent TTE at baseline and follow-up (Figure 1). This study population comprised 565 participants with available baseline TTE recruited in University of Navarra-Preventiva (Navarra, Spain), Hospital Universitario Araba (Vitoria, Spain), and Hospital Universitari Son Espases (Mallorca, Spain).

### Study Protocol and Setting

TTE tests will be used to longitudinally assess structural and functional features of the heart. TTE images were taken for each participant at baseline, and are expected to be taken at two timepoints of the follow-up (year 3 and 5 after the study entry, Figure 1). Data acquisition is performed according to the standards of the European Association of Echocardiography and the American Society of Echocardiography (ASE). Emphasis will be put into following the guidelines for evaluation of left atrial structure and function. Damage in left atrium might be the substrate for the development of atrial fibrillation (AF), among other disarrangements related to MetS. TTEs will be performed at each site following a standardized protocol (Table 1).

**TABLE 1:** Image acquisition protocol.

Item	View	Mode	Recording	Clip—frame
1	Paraesternal long axis	2D	Ao–RV–LV–mitral and aortic valve	3 Clips
2	Paraesternal long axis	2D color	Ao–RV–LV–mitral and aortic valve	3 Clips
3	Paraesternal long axis	2D	Epicardial fat	3 Clips
4	Paraesternal long axis	M Mode	Aortic valve–left atrium	Freeze frame
5	Paraesternal long axis	M Mode	LV–RV	Freeze frame
6	Paraesternal long axis	M Mode	Mitral valve tips	Freeze frame
7	Paraesternal short axis	2D, 2D color	Aortic–pulmonary–tricuspid valve–left atrium	3 Clips
8	Paraesternal short axis	2D	LV mitral level	3 Clips
9	Paraesternal short axis	2D	LV papillary muscle level	3 Clips
10	Paraesternal short axis	2D	LV apex	3 Clips
11	Paraesternal short axis	CW	Peak velocity of tricuspid regurgitation	Freeze frame
12	Paraesternal short axis	PW	RV outflow tract flow	Freeze frame
13	Apical 4 chambers	2D	Left and right ventricle and atrium	3 Clips
14	Apical 4 chambers	2D color	Mitral flow	3 Clips
15	Apical 4 chambers	PW	Mitral leaflets level inflow	Freeze frame
16	Apical 4 chambers	DTI–PW	Septal mitral annulus	Freeze frame
17	Apical 4 chambers	Color DTI	Left and right ventricle and atrium	Freeze frame
18	Apical 4 chambers	DTI–PW	Lateral mitral annulus	Freeze frame
19	Modified 4 Chambers	2D	View directed of the free wall of the RV	3 Clips
20	Apical 4 chambers	PW	Pulmonary vein flow	Freeze frame
21	Apical 4 chambers	Color M-mode	Mitral flow propagation velocity	Freeze frame
22	Apical 4 chambers	2D color	Tricuspid flow	3 Clips
23	Apical 4 chambers	CW	Peak velocity of tricuspid regurgitation	Freeze frame
24	Apical 4 chambers	M Mode	M mode tricuspid annulus (TAPSE)	Freeze frame
25	Apical 5 chambers	2D	LV and RV, left atrium, Ao–mitral and aortic valve	3 Clips
26	Apical 5 chambers	2D color	Aortic flow	3 Clips
27	Apical 5 chambers	2D color	Mitral flow	3 Clips
28	Apical 3 chambers	2D	LV and RV, left atrium, Ao–mitral and aortic valve	3 Clips
29	Apical 3 chambers	2D color	Aortic flow	3 Clips
30	Apical 3 chambers	PW	LV outflow tract	Freeze frame
31	Apical 3 chambers	CW	Aortic flow	Freeze frame
32	Apical 2 chambers	2D	LV and atria	3 Clips
33	Apical 2 chambers	2D color	Mitral flow	3 Clips
34	Subcostal view	2D	LV and RV and atrium	3 Clips
35	Subcostal view	2D	Inferior vena cava	3 Clips
36	Subcostal view	2D	Inferior vena cava	Freeze frame
37	Suprasternal view	2D color	Aortic arch flow	3 Clips

*Ao, aorta; RV, right ventricle; LV, left ventricle; CW, continuous wave, PW, pulsed wave; DTI, Doppler tissue imaging; TAPSE, tricuspid annular plane systolic excursion.*

The PEP will evaluate and measure all TTEs performed by the 3 participating centers using the Echopac version 204 post-processing tool, obtaining video clips of three beats per 2D projection and 15 s in the M-mode studies and Doppler and according to the protocol in Table 1. The CORELAB readers are two independent experts not involved in obtaining the images in any of the study sites, but only evaluating them. Primary and derived measures will be collected systematically, following a standardized protocol (Table 2).

**TABLE 2:** Transthoracic echocardiography measures.

Type of measures	Measures
Left atrial (LA) structure	LA anterior-posterior dimension, LA volume, LA area, LA volume index.
Left ventricular (LV) structure	LV end-diastolic dimension, LV end-systolic dimension, LV wall thickness, LV end-diastolic volume, LV end-systolic volume, LV mass, LV mass index, LV relative wall thickness.
Right ventricular (RV) structure	RV end-diastolic area, RV end-systolic area, RV free wall thickness.
Left atrial (LA) function	Passive LAEF, active LAEF, LA function index, LA stiffness index, LA longitudinal strain.
LV systolic function	Longitudinal strain, strain rate, radial strain, ejection fraction, standard deviation in time to peak longitudinal strain.
LV diastolic function	E wave, A wave, E/A ratio, E/E’ ratio, medial and lateral mitral annular velocity (e’), mitral deceleration time.
RV systolic function	Lateral systolic myocardial velocity, RV fractional area change, TAPSE.
Valvular function	Aortic valve: left ventricular outflow tract velocity-time integral, aortic valve peak velocity, mean gradient transvalvular. Mitral valve: mitral regurgitation jet area.
Pulmonary vascular function	Tricuspid regurgitation velocity, right ventricular outflow tract velocity-time integral, peak right ventricle (RV)–right atrium gradient, pulmonary vascular resistance.

*LA, left atrial; LV, left ventricular; RV, right ventricle; LAEF, left atrium ejection fraction; DTI, Doppler tissue imaging; TAPSE, Tricuspid annular plane systolic excursion.*

Quality of the image-records will be categorized as good, fair or poor. Good quality is when the structure or record under evaluation can be adequately identified and measured in all its aspects, whereas fair quality happens when the structures can be correctly observed, but some measurements are hard to obtain. Poor quality means that the study does not reach the standards to obtain reliable measurements.

### Data Recording and Transmission

Vivid E9 is be used to record TTE tests. Vivid E9 is a diagnostic ultrasound machine for cardiac diagnosis with Doppler physics and instrumentation, transducers are single crystal and matrix array. The following techniques are used to record images:2D and color imaging optimization, tissue Doppler, 2D strain/speckle tracking and Automated Function Imaging (AFI) 3D/4D Volume cardiovascular ultrasound technology. All images are transferred to a common laboratory image repository in the PEP core lab, to be evaluated by the two independent echo evaluators. Post-process analysis is performed with Echopac software, whereas CardioWorflow of General Electric is used to generate an easy-to-export data set for subsequent data management and statistical analyses.

### Image Acquisition Protocol and Techniques

**FIGURE 2:**
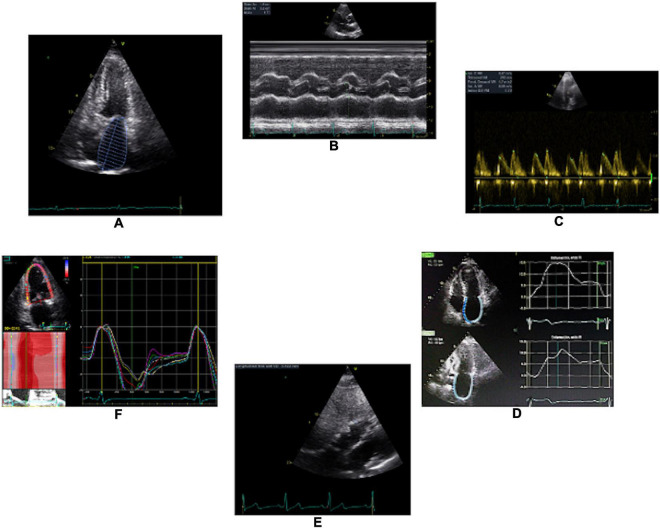
Illustration of some TTE parameters that will be recorded throughout a standardized protocol. **(A)**, left atrial size; **(B)**, left ventricle thickness; **(C)**, E and A waves; **(D)**, left atrium strain; **(E)**, right ventricular thickness; and **(F)**, left ventricular longitudinal strain.

Weight, height and blood pressure are routinely taken at the time of the TTE. Special attention would require the ECG signal (positive QRS). Imaging is recorded in unforced (non-Valsalva) apnea to avoid respiratory movement artifacts. For each view, ≥ 3 full cardiac cycles must be recorded. Raw data will be stored to allow for full post-processing capabilities (Echopac software) for 2D imaging. It is very important to maintain a frame rate of 60–90 frames per second (strain analysis). Color Doppler imaging Doppler Nyquist limit is set at 64 cm/s. For 2D Color TDI imaging is optimized with both imaging depth and sector width to maintain the frame rate as higher as possible. When recording time related display diagnostic techniques (e.g., spectral Doppler, M mode, DTI) the sweep speed used is 25–50 mm/s (not 100) to permit storing as many bytes as possible. If a relevant heart disease is documented during the echocardiographic evaluation (incidental finding), the protocol might be modified to further evaluate the participant. The Image Acquisition Protocol is presented in Table 2, and some illustrations are shown in Figure 2.

### Data Collection and Study Variables

In addition to the imagining data, some clinical data is needed to complete the assessment of participants with MetS. Information on age, sex, educational levels, medical history and family history was collected at baseline. Anthropometric evaluations (weight, height, waist circumference) were measured according to the PREDIMED-Plus protocol. BMI was calculated as weight (kg) divided by the square of height (m^2^). Further clinical data was collected at baseline, whereas additional data are becoming yearly available, such as food frequency questionnaires, adherence to the Mediterranean diet (surveyed with a 17-item questionnaire), physical activity measurements, blood pressure and electrocardiogram, cognitive neuropsychological tests (six tests every 2 years), the SF-36 (36-item) quality of life questionnaire (baseline and years 1, 3, 5, and 7), and three Psychopathological questionnaires. Further details can be found elsewhere (8). Moreover, some samples, such as plasma, serum, morning spot urine, DNA, and nails, are collected at baseline and years 1, 3, 5, and 7 of follow-up.

### Study Aims

The first hypotheses that is going to be tested through this platform will be the potential benefit of lifestyle interventions (erMedDiet, PA promotion and behavioral support) on the AF substrate in the subcohort of 565 participants with MetS from the PREDIMED-Plus trial. We hypothesize that the study intervention will avoid left atrial volume increase and improve left atrial function (assessed with novel measures of left atrial strain). Longitudinal TTE changes will be collected over a period of 5 years in participants from both the control and the intervention arm.

In addition to this initial hypothesis using randomized data, other broader hypotheses will be tested using the TTE data collected in the PEP. The overall aim of these subsequent observational studies will be to assess the association between the individual components of the MetS and some relevant TTE parameters measured either at a cross-sectional-level (baseline), or at longitudinal-level (5 years). Changes or alterations in the cardiac anatomy and function will shed light in the pathophysiology underlying the MetS. This evidence may be useful for the early detection of subjects with very high CV risk who might benefit from an early or intensive intervention.

### Data Management

STATA software version 15.1 (Stata Corp., College Station, TX, United States) and GraphPad Prism version 6.00 (GraphPad Software, La Jolla California, United States) will be used to perform the analyses and produce the graphs, respectively. The results will be adequately reported according to the research reporting guidelines for observational studies, or randomized controlled trials (the STROBE or CONSORT statement, respectively) (9, 10). The interpretation of results will emphasize estimates of effect, precision of those estimates (confidence intervals), and clinical significance rather than *p*-values and statistical significance (11).

## Discussion

The PEP is a highly coordinated network that has been built up to evaluate the underlying structural and functional cardiac substrate of participants with MetS. Although the information provided by TTEs can be used to answer many research questions, this platform is going to initially test for the potential benefit of lifestyle interventions (erMedDiet, PA promotion, and behavioral support) on the AF substrate in a subcohort of 565 participants with MetS from the PREDIMED-PLUS trial. In addition to this study using randomized data, further subsequent studies will focus on the impact of the individual components of the MetS (e.g., hypertension) on relevant echo markers based, regardless of the interventional allocation. Eventually, clinical outcomes, such as incident AF, ischemic heart disease, and heart failure, will become available to assess the prognostic importance of these echo markers. Given that the individual components of the MetS are risk factors for the development of ischemic heart disease and ventricular systolic and diastolic dysfunction, it is expected that participants with MetS will progressively experience changes in both the coronary arteries and the heart structure, which would precede the development of these clinical outcomes (12). Hence, the longitudinal use of TTE markers to better phenotype and screen patients with MetS represents a good opportunity to both understand the underlying mechanisms of myocardial impairment as well as to identify those patients with MetS who might benefit from an early preventive intervention (13).

The atrium has been traditionally evaluated in its morphological dimension (e.g., atrial volume). In our study, we included novel parameters that may provide relevant information, like left atrial strain and atrial ejection fraction. Traditionally, the function of the left atrium has been studied with parameters surrogated to blood flow during atrial contraction such as the peak A wave velocity or the A wave velocity time integral (14). Furthermore, these parameters are applicable only in sinus rhythm. Left atrial strain represents a direct measure of atrial function and has already been shown to alter earlier than atrial volume in healthy aging (14).

The initial hypotheses that will be tested through this platform will be the potential benefit of lifestyle interventions on the AF substrate in a population with MetS. AF is a common cardiac arrhythmia associated with an increased risk of stroke, heart failure, dementia, and cardiovascular mortality (15, 16). In a large American study, 22% of visits to the emergency department were for AF, and this figure has increased by 30.7% from 2007 to 2014 (17). Current treatments for AF, including antiarrhythmics, oral anticoagulants, and catheter ablation, have limited efficacy and involve significant risks, highlighting the need for prevention strategies (16). Unfortunately, there is little evidence of effective interventions preventing the onset of AF. Previous observational studies have shown that increased body weight is a major risk factor for the development of AF (18). There is an increasing body of evidence suggesting that this association might be mediated through a number of pathways, including alterations of the epicardial adipose tissue biology, activation of inflammatory pathways, development of structural cardiac remodeling, as well as atrial fibrosis. Importantly, weight loss in patients with AF has been associated with a slower progression from paroxysmal to persistent AF, and with a regression from persistent to paroxysmal AF (19). Data from the PREDIMED study has shown that individuals taking a Mediterranean diet enriched in extra-virgin olive oil had lower risk of AF than those receiving a control diet (20). Changes in the atrial substrate are likely to be responsible for the association between obesity, MetS and AF (21, 22), and imaging tests might be helpful to understand the pathophysiological mechanisms underlying the effect of diet and other lifestyle interventions, such as the PREDIMED-Plus intervention (a weight loss intervention program based on an erMedDiet, PA promotion and behavioral support), in AF-related outcomes. The potential effect weight loss on AF has been also studied in patients who already had AF. The ARREST-AF cohort study found that CVRF management, including weight loss, in AF patients undergoing ablation was associated with lower risk of recurrence (23). Using the LEGACY cohort, Ninomiya et al. reported that long-term (> 2 years) weight loss was associated with arrhythmia-free survival and significant changes in echocardiography-assessed left atrial structure (24). Given that these studies were performed on patients with AF, they were unable to address the role that weight loss interventions would have in the primary prevention of AF. The PEP will be suitable to response the scientific question about the potential efficacy of an intensive lifestyle intervention on the AF substrate, measured by TEE.

Although left ventricular ejection fraction and left atrial volume are commonly used to evaluate the mechanics of ventricular contraction, they have some limitations. One of the strongest points of our study is the systematic evaluation of left ventricular longitudinal strain. The heart is a single muscular band that folds over itself in the form of a double spiral, generating three different types of fibers (longitudinal, oblique, and transverse, located in subendocardium, subepicardium, and between both of them, respectively). In ventricular systole, subepicardial, and subendocardial fibers contract at different times and in the opposite direction to achieve an efficient contraction (25). Left ventricular longitudinal strain is a function parameter of subendocardial muscle fibers, which are the first fibers to be affected in systolic ventricular dysfunction (26). Hence, left ventricular longitudinal strain is useful for the detection of subclinical systolic dysfunction, and has been shown to be a sensitive, reproducible parameter with less variability than the left ventricular ejection fraction (27). The information provided by left ventricular longitudinal strain will be useful to phenotype the myocardium in participants with MetS.

PEP will ensure a well-structured platform interpreting echo data in a reliable and reproducible manner. Previous experiences from other randomized studies, such as the ISCHEMIA (International Study of Comparative Health Effectiveness with Medical and Invasive Approaches) and the PARTNER I (Placement of Aortic Transcatheter Valves) trials, have shown the usefulness of setting an echocardiography Core labs to reduce variability and obtain representative and comparable values across participants over time (28–30).

### Limitations

This is a nationwide randomized controlled study, and caution should be taken when extrapolating our potential findings to a real-world population. The quality of the TTE acquisition does not only rely on the evaluator, but on the window of participating subjects, which is particularly challenging in a population with a high body mass index and MetS. Other techniques, such as cardiac magnetic resonance, might be more precise to define tissue characterization and other myocardial parameters (31). Moreover, other echo parameters might be studied in *post hoc* analyses, such as the maximal epicardial adipose tissue (EAT) thickness at the Rindfleisch fold, measured from parasternal long axis 2D imaging at diastole. Sample size was calculated to estimate the effects of a lifestyle intervention on echo parameters known to be associated with later risk of AF in a sub-sample, taking into account the longitudinal assessment (repeated measures) and multiple testing of echocardiographic parameters. Any association between non-randomized data (such as echo parameters) and CV events (e.g., AF) would be hypothesis-generating.

## Conclusion

In this article, we described the protocol of a central echocardiography laboratory (PEP) to assess the myocardial substrate of participants with overweight or obesity and MetS. Repeated TTE performed at three study sites will be used to evaluate 5-year changes in the cardiac structure and function in a group of 565 individuals participating in a randomized trial of a lifestyle intervention for the primary prevention of cardiovascular disease. Planned analyses will involve evaluating the effect of the lifestyle intervention on cardiac structure and function, and the association of the MetS and its components with changes in cardiac structure and function. Particular emphasis will be placed on evaluating parameters of left atrial structure and function, which have received little attention in previous investigations.

## Author Contributions

DR, MF, AA, and CF-P: conception and design. LL, XR, and DR: drafting of the article. AA, ET, ÁA-G, and DR: obtaining of funding. LL, ÁA-G, ET, EF, MN, CM-L, RR, and LT-S: collection and assembly of data. All authors: critical revision and final approval of the article.

## Author Disclaimer

The content was solely the responsibility of the authors and does not necessarily represent the official views of the National Institutes of Health.

## Conflict of Interest

The authors declare that the research was conducted in the absence of any commercial or financial relationships that could be construed as a potential conflict of interest.

## Publisher’s Note

All claims expressed in this article are solely those of the authors and do not necessarily represent those of their affiliated organizations, or those of the publisher, the editors and the reviewers. Any product that may be evaluated in this article, or claim that may be made by its manufacturer, is not guaranteed or endorsed by the publisher.
